# Comparing aerosol number and mass exhalation rates from children and adults during breathing, speaking and singing

**DOI:** 10.1098/rsfs.2021.0078

**Published:** 2022-02-11

**Authors:** Justice Archer, Lauren P. McCarthy, Henry E. Symons, Natalie A. Watson, Christopher M. Orton, William J. Browne, Joshua Harrison, Benjamin Moseley, Keir E. J. Philip, James D. Calder, Pallav L. Shah, Bryan R. Bzdek, Declan Costello, Jonathan P. Reid

**Affiliations:** ^1^ School of Chemistry, University of Bristol, Bristol, UK; ^2^ School of Education, University of Bristol, Bristol, UK; ^3^ Department of Ear, Nose and Throat Surgery, Guy's and St Thomas' NHS Foundation Trust, London, UK; ^4^ Department of Respiratory Medicine, Royal Brompton Hospital, London, UK; ^5^ Department of Respiratory Medicine, Chelsea and Westminster Hospital, London, UK; ^6^ National Heart and Lung Institute, Imperial College London, Guy Scadding Building, London, UK; ^7^ Department of Bioengineering, Imperial College London, London, UK; ^8^ Fortius Clinic, Fitzhardinge Street, London, UK; ^9^ Ear, Nose and Throat Department, Wexham Park Hospital, Slough, UK

**Keywords:** aerosol, respiratory pathogens, airborne transmission, exhalation

## Abstract

Aerosol particles of respirable size are exhaled when individuals breathe, speak and sing and can transmit respiratory pathogens between infected and susceptible individuals. The COVID-19 pandemic has brought into focus the need to improve the quantification of the particle number and mass exhalation rates as one route to provide estimates of viral shedding and the potential risk of transmission of viruses. Most previous studies have reported the number and mass concentrations of aerosol particles in an exhaled plume. We provide a robust assessment of the absolute particle number and mass exhalation rates from measurements of minute ventilation using a non-invasive Vyntus Hans Rudolf mask kit with straps housing a rotating vane spirometer along with measurements of the exhaled particle number concentrations and size distributions. Specifically, we report comparisons of the number and mass exhalation rates for children (12–14 years old) and adults (19–72 years old) when breathing, speaking and singing, which indicate that child and adult cohorts generate similar amounts of aerosol when performing the same activity. Mass exhalation rates are typically 0.002–0.02 ng s^−1^ from breathing, 0.07–0.2 ng s^−1^ from speaking (at 70–80 dBA) and 0.1–0.7 ng s^−1^ from singing (at 70–80 dBA). The aerosol exhalation rate increases with increasing sound volume for both children and adults when both speaking and singing.

## 1. Introduction

The coronavirus disease (COVID-19) pandemic continues to heighten awareness of the potential for aerosols and droplets to transmit respiratory pathogens, including the severe acute respiratory syndrome coronavirus-2 (SARS-CoV-2). Respiratory aerosols and droplets can be generated by an infected individual during respiratory activities like breathing, speaking and singing [1–4]. In particular, person-to-person transmission of the highly transmissible SARS-CoV-2 can occur by inhalation of pathogen-laden aerosol particles from an infected person into the respiratory tract of a susceptible individual in close proximity, [5–9], or at a far distance in a poorly ventilated or an enclosed space [10,11]. Breathing, speaking, singing, coughing and sneezing generate droplets and aerosols of varying size, concentration and viral load [4,12–15]. The delineation between aerosols, droplet nuclei and droplets is often imprecise with somewhat arbitrary distinctions made between respirable aerosols (less than or equal to 5 µm diameter), inhalable particles (less than or equal to 100 µm) and large droplets (greater than 100 µm) [16–18]. Indeed, Prather *et al*. have highlighted that all particles < 100 µm exhibit similar aerodynamic behaviour and can be dispersed beyond the typical distances assumed by physical distancing guidance [19]. Based on this more nuanced recognition of the size-dependent aerodynamic properties of droplets and aerosols [20,21], Marr & Tang [6] have recently suggested that the size delineation between droplets and aerosols should be more appropriately set at 100 µm. Particles of approximately 100 µm in diameter represent the largest size that is inhalable and can remain suspended in still air for greater than 5 s, travelling beyond 1 m in the exhaled plume from a infectious person [6,22]. In fact, SARS-CoV-2 transmission through the inhalation of these aerosol and spray droplets is considered to play a significant role in super-spreading events that involved close contacts and group activities [22,23]. In addition, although ballistic droplets produced by an infectious individual through coughing or sneezing are capable of transporting infectious pathogens over metres, recent studies have suggested that the viral load and number concentration of aerosol particles less than or equal to 5 µm in diameter produced by speaking and other expiratory activities could be much higher than associated with larger particles [22,24–26]. Numerous COVID-19 outbreaks among both children and adults are now thought to be linked to airborne transmission [5], including the Skagit Valley chorale rehearsal [27,28].

Previous studies have focused on quantifying expiratory aerosols emitted from adults during breathing, speaking, singing and coughing [1–4] with more limited studies on adolescent or pre-adolescent children [20]. Children and adolescents are equally susceptible to infection with SARS-CoV-2 [21] and can transmit the virus to others [22,29]. By contrast to adults, the SARS-CoV-2 infection is usually more benign in children, with a greater proportion asymptomatic or showing milder symptoms, and with significantly lower mortality rate than in adult infections [23,30]. Despite lower rates of hospitalization and mortality in children, concerns persist over the rates of transmission in classrooms and in activities, such as singing.

Previous studies by ourselves and others have quantified the absolute particle number and mass concentrations of aerosols in exhaled air, an *intensive* property, or the relative particle exhalation rates from different activities through comparing detected particle numbers [1–4,13,20,31–33]. In this paper, we quantify and compare absolute source-specific respiratory aerosol particle exhalation rates from children and adults during expiratory activities, an *extensive* property. *Intensive* properties (e.g. temperature, concentration) do not depend on the system size and should be contrasted with *extensive* properties that do depend on system size (e.g. mass, volume). In the context of respiratory aerosol, the *extensive* property of particle exhalation rate provides an absolute assessment of the aerosol generation rate that can be used to estimate absolute amounts of virus shed by an individual and is a much more appropriate quantity when considering the risks associated with transmission. More specifically, we combine a non-invasive Vyntus Hans Rudolf mask kit with straps housing a rotating vane spirometer for minute ventilation measurements with detected aerodynamic particle sizes and concentrations to report the *absolute* number and mass exhalation rates of aerosol particles produced during breathing, speaking and singing by children and adults. Our first aim is to explore the variability in number concentration of expired aerosol particles generated during breathing and vocalization by children compared to a wider cohort of adults. We further consider aerosol particle number and mass concentrations, minute ventilations, exhalation rates (number and mass) and size distributions of aerosol particles (approx. 0.5–20 µm) from breathing, speaking, singing and sustained vocalization (/a/) across cohorts of healthy children and adults performing similar expiratory activities. Finally, we will assess contributing factors in respiratory aerosol generation based on loudness of vocalization and minute ventilations, and their linked dependence on the number exhalation rates of expelled aerosol particles.

## 2. Methods and study design

### 2.1. Human participants

As part of the PERFORM-2 project and through contact and collaboration with school choirs in England, we recruited 18 healthy children volunteers (nine male and nine female), ranging in age from 12 to 14 years with a (mean ± standard deviation, median) of (13.1 ± 0.7, 13.2); males (13.7 ± 0.7, 13.5), females (12.9 ± 0.6, 12.7). Informed consent was obtained from parents and guardians who were present at the time of the measurement procedures. We also recruited 118 healthy adult volunteers across PERFORM-1 and -2, and AERATOR studies (58 male and 60 female) ranging in age from 19 to 72 years old (40.9 ± 12.2, 38.0). All children and adults were pre-screened to ensure they were healthy, which was defined as free from cardiac, metabolic, or respiratory disease, including severe asthma and COVID-19 symptoms. We also ensured that both the children and adults completed a pre-screening questionnaire including questions regarding age, gender, weight, height, singing training history and ethnicity to fulfil inclusion/exclusion criteria.

### 2.2. Speaking and singing vocalization experiments

Participants performed voiced and unvoiced activities similar to our earlier protocols for adult professional singers, instrumentalists and amateurs as reported in our previous studies [4,31]. Briefly, participants performed a series of five repeated sustained vocalizations of ‘/a/’ (the vowel sound in ‘far’) each for 10 s at a target volume of 70–80 dBA. Between each repeat, subjects stepped away from the sampling funnel for 20 s to ensure the measured aerosol concentration reduced to background levels (0 cm^−3^). The participants also performed a confirmatory ‘/a/’ experiment at the end of the session to ensure reproducibility of the measurement. Participants then performed a series of three speaking and three singing experiments using the words of the ‘Happy Birthday’ song addressing ‘Dear Susan’, each for 20 s followed by 30 s at rest stepping away from the sampling funnel. The three sets of measurements for speaking and singing were made at volumes of: 50–60 dBA and 60–70 dBA as the quietest volume for adults and children, respectively; 70–80 dBA for both child and adult cohorts; and 80–90 dBA for children and 90–100 dBA for adults. Voice amplitudes and sound level meter readings were recorded concurrently with 1 s samples for both speaking and singing activities.

### 2.3. Breathing experiments

Participants breathed for 10 s inhaling through the nose and exhaling through an open mouth in a non-forced ‘quiet’ fashion, standing 2 m away from the funnel for 30 s in between each repeated measurement. This activity was repeated 5 times in total.

### 2.4. Measurements of respired aerosol concentrations and vocal loudness

The expired aerosols generated from the different activities were measured following the same experimental set-up configuration and procedures used in our previous studies [4,31]. Briefly, an aerodynamic particle sizer (APS 3321 from TSI Incorporated, MN, USA, sampling at 1 l min^−1^ with sheath flow of 4 l min^−1^) measured expired aerosols (0.5–20 µm) sampled via a collection funnel and through a 100 cm section of conductive tubing (TSI Inc., inner diameter 0.19 inch, outer diameter 0.375 inch). The conductive tubing was carefully straightened to minimize bends and avoid tight curvatures, always maintaining a ratio of the radius of curvature (*r*_c_) to the inner tube radius (*r*_t_) greater than 50. A Datalogger Sound Level Meter with an LCD display screen (RS PRO RS-8852 Sound Level Meter, accuracy ±1.4 dB, dynamic range 30–130 dB, resolution 0.1 dB) was mounted at approximately 30–40 cm from the sampling funnel and at an adjustable height with the display visible to the participant eye level to simultaneously record their voice amplitude in dB allowing them to self-regulate their voice amplitudes. The sampling frequency of the sound level meter was set at 1 s to match the aerosol measurement sampling rate on the APS.

**Figure 1.**
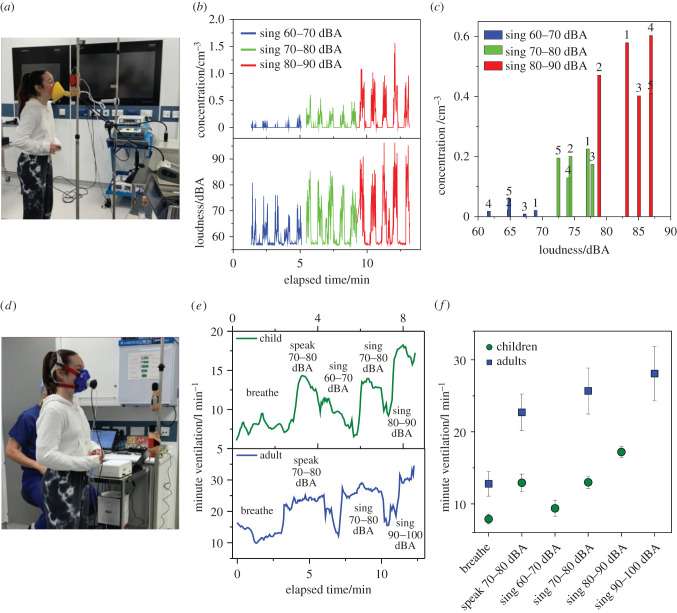
Experimental configuration of (*a*) APS aerosol measurements and (*d*) minute ventilation measurements. (*b*) Continuous time series for one participant completing three activities (five repetitions of each) showing number concentration (cm**^−^**^3^, top panel) and loudness (dBA, bottom panel). (*c*) Averaged number concentration (cm**^−^**^3^) for each repeat of three activities and the corresponding loudness measured. (*e*) Continuous time series for individual participants (child, top panel; adult, bottom panel) completing various activities showing minute ventilation (l min**^−^**^1^). (*f*) Averaged minute ventilation (l min**^−^**^1^) for each activity across each cohort of adults and children.

All the measurements were carried out in a laminar flow operating theatre, with a near-zero background aerosol number concentration in the 0.5–20 µm size range, allowing quantification of the relatively small amounts of respiratory particulate matter produced from the expiratory activities [4]. The aerosol measurement configuration is shown in figure 1*a*. A representative time series recording of raw aerosol particle number concentration with corresponding sound pressure data for a single child participant performing a series of five successive repeats of singing at 60–70 dBA, 70–80 dBA and 80–90 dBA is shown in figure 1*b*. Figure 1*c* reports the time-averaged aerosol number concentration of the five successive repeats of the singing exercise with corresponding logarithmic average sound pressure at which the activities were performed. It also shows a relation between increase in loudness of continuous vocalization with the concentration of emitted particles.

### 2.5. Respiratory airflow measurements during breathing, speaking and singing

We used a non-invasive reusable Vyntus Hans Rudolf mask kit (Hans Rudolph 7450V2, complete with adapter and headgear; size: petite and medium, Vyaire Medical GmbH) with straps housing a rotating vane spirometer for measurements of minute ventilation (an airflow rate in units of l min^−1^) of participants performing breathing, speaking and singing activities. Upon familiarization with the kit, the participant wore the mask as in figure 1*d*, in a manner that did not inhibit the free movement of the jaw or distort higher frequency sounds during speaking or singing. Sound levels were recorded using the Datalogging Sound Level Meter at 1 s sampling rate. Each child participant initially breathed for 3 min then spoke ‘Happy Birthday’ at 70–80 dBA for 60 s. This was followed by 30 s rest then singing ‘Happy Birthday’ at 60–70 dBA for 60 s. The participant then sang ‘Happy Birthday’ at 70–80 dBA followed by 80–90 dBA, each for 60 s with a 30 s pause (at rest) between the two volumes. Similarly, minute ventilation measurements were also performed for eight adult singers undertaking similar activities. Each adult, after familiarization with the kit, wore the mask and breathed for 3 min. At the end of the breathing, the participant spoke ‘Happy Birthday’ for 3 min at 70–80 dBA followed by 60 s pause. The participant then sang ‘Happy Birthday’ for 3 min at 70–80 dBA and 60 s at 90–100 dBA with a 60 s pause between the two events. We note here that similar studies by us [34] on sampling aerosol emissions directly through the Hans Rudolf mask in cardiopulmonary exercise testing (CPET) across 25 adult participants performing different exercise activities found minimal influence on the size and concentrations of the expelled aerosols measured. The mean number (*p* = 0.152) and mass concentrations (*p* = 0.060) of emitted aerosols measured inside the CPET mask via a tube to an APS during speaking at a level of 70–80 dBA were very comparable with our previously reported measurements on 25 adult singers during speaking at the same sound level [4]. Similarly, the average size distributions were also comparable within the error bounds of previously reported measurements [4,33].

Figure 1*e* compares characteristic time series of minute ventilation measurements recorded for a child and an adult participant during breathing, speaking and singing at different volumes. Figure 1*f* represents the time-averaged minute ventilation from the time series data reported in figure 1*e*.

### 2.6. Data processing and statistical analysis

The raw data of aerosol counts from the APS instrument were collected with Aerosol Instrument Manager software (TSI, USA) and post-processed with a custom-written software in LabVIEW as described in Gregson *et al*. [4]. The post-processed files were then analysed in Origin (OriginLab). For the statistical analysis, variables were aggregated to the individual level due to different sampling regimes across studies. Data were inspected and log transforms were used when the data were skewed. For pairwise comparisons between adults and children, independent sample *t*-tests were used while for comparisons of different activities within individuals paired *t*-tests were used. To account for the multiple hypotheses performed (43 tests) in this paper, we performed a false discovery rate adjustment. Using an alpha of 0.05, this suggests adjusting the threshold of significant results from 0.05 to 0.03. This removes one significant effect, specifically, the comparison of singing a single note adult versus child for particle number concentration with a *p*-value of 0.038.

## 3. Results and discussion

Initially, we will compare the intensive property of aerosol concentration measured in the exhaled plume from children and adults breathing, speaking and singing, and consider the consistency with a previously published study of participants breathing [35]. After comparing aerosol particle size distributions generated during breathing, speaking and singing by adults and children, we introduce estimates of the extensive property of aerosol particle number and mass exhalation rate, again comparing with estimates from previous studies. Finally, we explore the relationship between the aerosol number exhalation rate and the loudness of the vocalization and minute ventilation (air flow rate).

### 3.1. Comparison of aerosol number concentrations generated by children and adults while breathing and vocalizing

**Figure 2.**
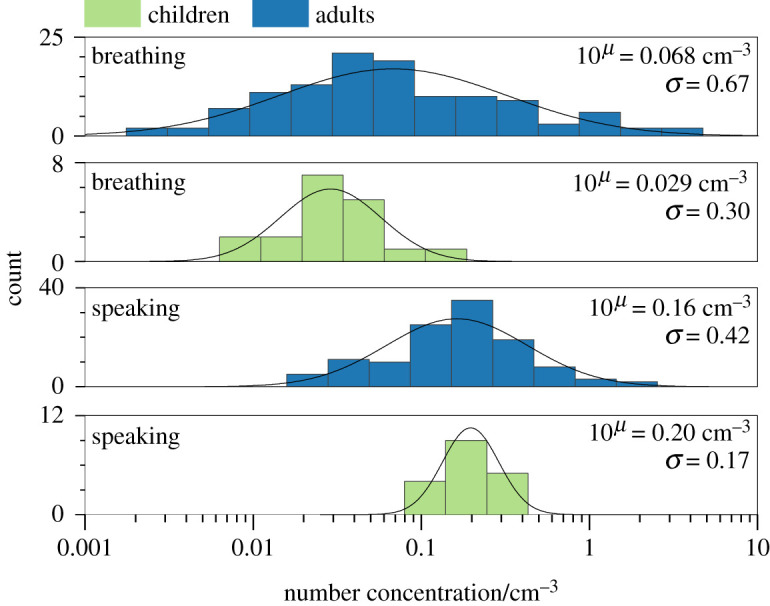
Histograms of aerosol particle number concentration from adults (*n* = 118) and children (*n* = 18) while breathing and speaking at 70–80 dBA. Histogram bin widths are equal in log(number concentration). The distribution is a lognormal fit to the data. *μ* represents the geometric mean of the lognormal - distributions, whereas *σ* represents the width of the lognormal distribution.

Figure 2 compares the ranges of aerosol number concentrations generated by children (*n* = 18) and adults (*n* = 118, aggregate cohort across PERFORM and AERATOR studies) [4,31,34,36] while breathing and speaking at 70–80 dBA. For both breathing and speaking and across both cohorts, differences in aerosol generation among individuals are lognormally distributed, consistent with previous comparisons of aerosol generation across smaller cohorts [4,31]. Lognormal distribution parameters are provided in electronic supplementary material, table S1. Quantitative analysis of these lognormal distributions provides insight into the mean number concentration generated and the standard deviation across participants (*σ*). Breathing exhibits a very broad distribution across individuals: in adults, aerosol generation spans over four orders of magnitude (*σ* = 0.67), whereas for children it spans approximately two orders of magnitude (*σ* = 0.30). The broad distribution observed for adults is consistent with observations by Edwards *et al*. [35] (*n* = 194), with the range of the distributions from the current and previous study matching almost exactly (see electronic supplementary material, figure S1).

By contrast to breathing, speaking generates a significantly narrower range of aerosol concentrations: aerosol generation spans only 2–3 orders of magnitude (*σ* = 0.42) in adults and approximately one order of magnitude (*σ* = 0.17) in children. Number concentrations generated while speaking are significantly higher than those generated while breathing (*p* < 0.001 for both the child and adult cohorts). The distributions for adults shift from a mean concentration of 0.068 cm^−3^ while breathing to 0.16 cm^−3^ while speaking, whereas for children the distributions shift from 0.029 cm^−3^ while breathing to 0.20 cm^−3^ while speaking, consistent with previous observations [1,3,4,31]. There is a small but statistically significant difference between the child and adult cohorts breathing (*p* < 0.001). However, the range of the child cohort is encompassed by that of the adult cohort, and the difference could be an artefact of the much larger adult cohort size. For speaking, no significant difference is observed between the child and adult cohorts (*p* = 0.145). Further examination of the number concentration emitted during breathing with participant age (from 12–72 years) shows no significant difference with age (see electronic supplementary material, figure S2).

### 3.2. Comparison of aerosol number and mass concentrations from children and adults during breathing, speaking and singing

We now compare aerosol number and mass concentrations generated by the child and adult cohorts across a wider range of respiratory activities that include vocalizing (speaking and singing) at multiple sound volume levels. The mass concentrations are inferred from size-resolved measurements of particle number concentration, assuming a particle density equal to that of water (1 g cm^−3^) in accordance with our previous publications [4,31]. Alternatively, the size distribution could be reflected by reporting a volume concentration; a mass concentration of 1 µg m^−3^ is equivalent to a volume concentration (volume of condensed phase per unit volume of gas phase) of 1 × 10^–6^ cm^3^ m^−3^ using an assumed density of 1 g cm^−3^. A full examination of the size distributions is presented in §3.3. The cohort size for children remains the same as in §3.1 (*n* = 18). However, the cohort size for adults varies by activity (*n* spans 32–118 participants) as different sub-cohorts of adults performed different activities [4,31,34]. Our previous publication noted no resolvable differences in aerosol concentrations across gender for an adult cohort, [4] and we note the same observation for the cohort of children (see electronic supplementary material, figure S3).

**Figure 3.**
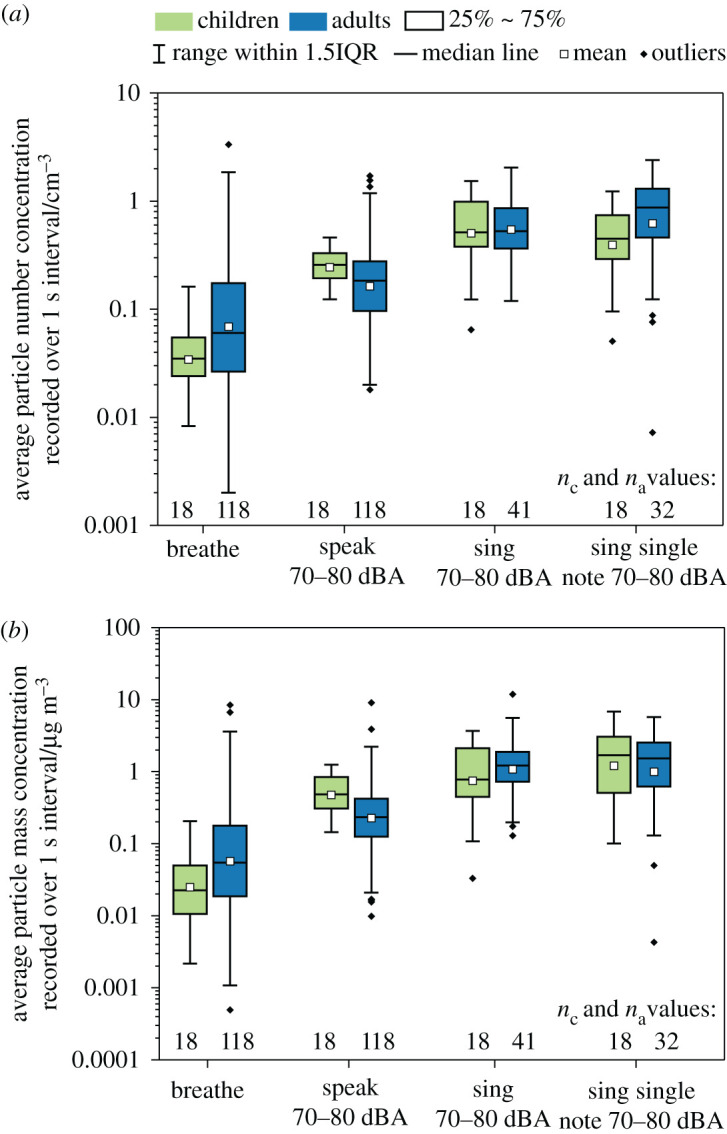
(*a*) Number and (*b*) mass concentrations of aerosols generated from breathing, speaking and singing at 70–80 dBA and sustained vocalization of /a/ at 70–80 dBA. *n*_c_ and *n*_a_ are the number of participants for each activity in the child and adult cohorts, respectively.

Figure 3 presents box and whisker plots for aerosol number concentration (figure 3*a*) and mass concentration (figure 3*b*) generated during respiratory activities in both the child and adult cohorts: breathing, speaking at 70–80 dBA, singing at 70–80 dBA, and singing a single note (/a/) at 70–80 dBA. The data in the figure are also summarized in table 1.

**Table 1.** Summary of the measured aerosol number and mass concentrations from different expiratory activities for children and adults. Number concentration (in cm^−3^) data correspond to the series of expiratory activities plotted in figure 3*a*, and mass concentration (in µg m^−3^) data correspond to the series of expiratory activities plotted in figure 3*b*. The number of participants for each activity is given for both the children and the wider cohort of adults.

	parameters	activities							
		breathe		speak 70–80 dBA		sing 70–80 dBA		sing single note 70–80 dBA	
		children	adults	children	adults	children	adults	children	adults
particle number concentration (cm^−3^)	mean	0.029	0.069	0.20	0.16	0.40	0.55	0.31	0.62
	median	0.029	0.060	0.21	0.18	0.41	0.53	0.36	0.87
	25%	0.02	0.026	0.16	0.096	0.30	0.36	0.23	0.46
	75%	0.046	0.17	0.26	0.28	0.77	0.86	0.58	1.3
	bottom whisker	0.0072	0.0020	0.10	0.018	0.053	0.12	0.042	0.0072
	top whisker	0.13	3.3	0.36	1.7	1.2	2.0	0.95	2.4
	*n*	18	117	18	118	18	41	18	32
particle mass concentration (µg m^−3^)	mean	0.025	0.057	0.48	0.23	1.2	1.1	0.75	0.99
	median	0.023	0.055	0.49	0.23	1.7	1.2	0.78	1.5
	25%	0.011	0.019	0.31	0.13	0.51	0.73	0.45	0.62
	75%	0.050	0.18	0.84	0.42	3.1	1.9	2.1	2.5
	bottom whisker	0.0022	0.00050	0.14	0.0098	0.10	0.13	0.033	0.0043
	top whisker	0.21	8.4	1.2	9.1	6.8	12	3.7	5.7
	*n*	18	117	18	118	18	41	18	32

The child and adult cohorts generated similar number concentrations while speaking (*p* = 0.147) and singing at 70–80 dBA (*p* = 0.127). Relatively small (factor of < 2.5) but statistically significant differences in number concentrations were observed between the child and adult cohorts while breathing (*p* < 0.001, as discussed in §3.1) and while singing a single note (*p* = 0.038). Notably, within each cohort, the aerosol number concentration generated while singing ‘Happy Birthday’ at 70–80 dBA was similar to that generated by singing a single note (*p* = 0.293 and *p* = 0.420 for child and adult cohorts, respectively), suggesting that the aerosol number concentration generated by singing at a specific loudness is not especially dependent on the type of song. By contrast, within an individual cohort, statistically significant differences between breathing and speaking (*p* < 0.001 for both child and adult cohorts) and between speaking and singing (*p* < 0.001 for adults and *p* = 0.003 for children) were observable. As observed in our previous work [4], the differences between speaking and singing are modest (a factor of 2–3 in particle number concentration).

A comparison of mass concentrations emitted by children and adults for breathing, singing and sustained vocalization showed no significant variation between the two cohorts (*p* = 0.082, *p* = 0.702 and *p* = 0.493, respectively). There was a small (factor of 2) but statistically significant difference in aerosol mass concentration between the child and adult cohorts during speaking (*p* = 0.012). Intra-cohort comparisons of the mass concentrations generated while singing and sustained vocalization found no statistical difference for either the child or adult cohorts (*p* = 0.128 and *p* = 0.895, respectively). As with number concentration, statistically significant differences between breathing and speaking (*p* < 0.001 for both child and adult cohorts) and between speaking and singing (*p* < 0.001 for adults and *p* = 0.006 for children) were observed within both cohorts. However, the differences between speaking and singing are modest compared to the difference between breathing and speaking. Overall, these comparisons indicate that children and adults generate similar aerosol concentrations when performing the same activity and that vocalization generates significantly more aerosol than breathing.

**Figure 4.**
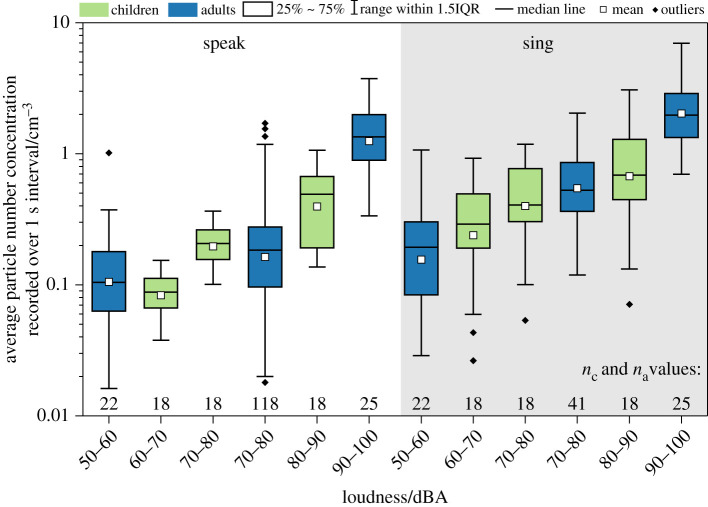
Number concentrations measured from speaking (non-shaded) and singing (shaded) at different levels of loudness for both the child and adult cohorts. *n*_c_ and *n*_a_ are the number of participants for each activity in the child and adult cohorts, respectively.

The conclusion that vocalization is an important driver of aerosol emission is emphasized in figure 4, which shows number concentrations measured during vocalization (both speaking and singing) at different sound volumes for the child and adult cohorts. The data in the figure are also summarized in electronic supplementary material, table S2. A clear sound volume dependence is observed, with exhaled number concentration increasing with increasing loudness for both children and adults. Our previous study noted a statistically significant difference between speaking at 50–60 dBA and 90–100 dBA as well as between singing at 50–60 dBA and 90–100 dBA for adults [4]. We note the same observation for children, with statistically significant differences between speaking at 60–70 dBA and 80–90 dBA (*p* < 0.001) and between singing at 60–70 dBA and 80–90 dBA (*p* < 0.001) for children. This observation is consistent with previous studies noting a volume dependence for vocalization and instrument playing [1,4,31].

### 3.3. Aerosol size distributions generated by children and adults while vocalizing

**Figure 5.**
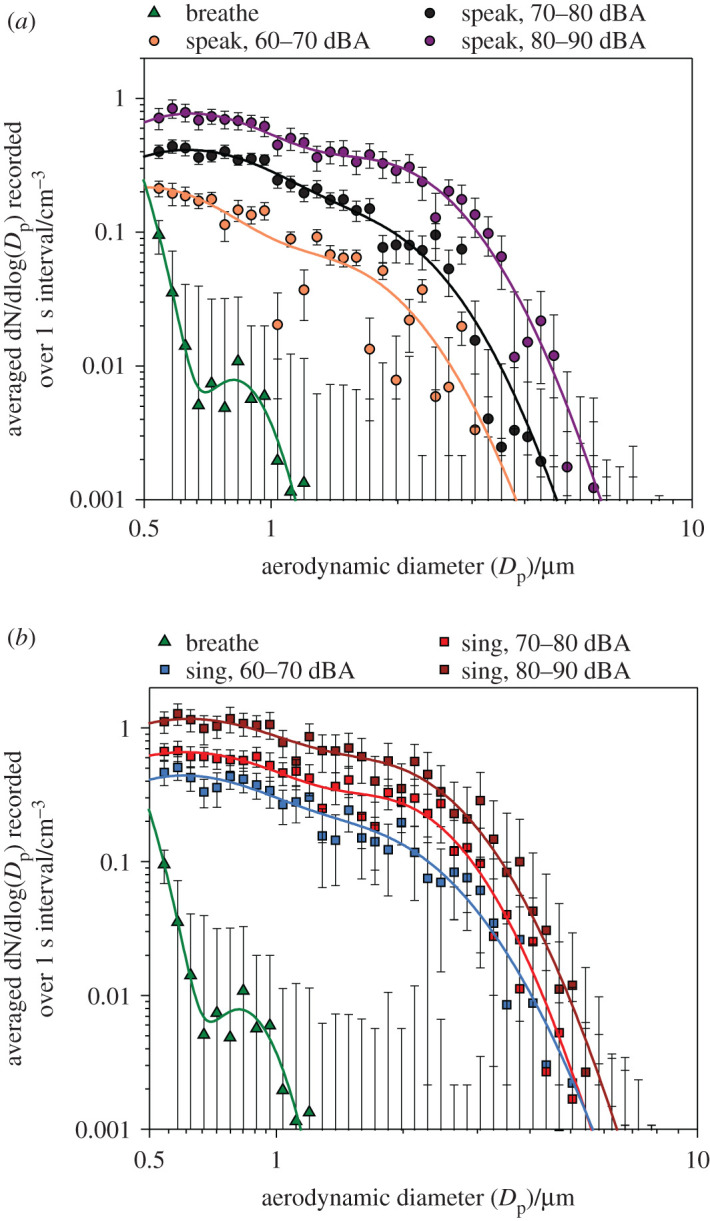
Comparison of mean aerosol size distributions generated by children when breathing and (*a*) speaking and (*b*) singing at different sound volume levels. Curves of the corresponding colour indicate bimodal lognormal fit of each dataset. Error bars represent standard error of the mean.

Figure 5 shows mean size distributions across the entirety of the child cohort for breathing and for speaking and singing at each volume range studied. In agreement with previous reports on respiratory aerosol, size distributions for all activities could be fitted using bimodal lognormal distributions (*R*^2^ > 0.89). Full fitting parameters for all size distributions are provided in electronic supplementary material, table S3.

For all activities involving vocalization, the mode of smaller particle size was centred around 0.50–0.64 µm diameter, indicative of particles generated within the lower respiratory tract [13,32,33]. The larger-sized mode was between 1.39 and 1.94 µm diameter during vocalization, representative of particles formed in the larynx. Although the mean breathing size distribution could also fit to a bimodal distribution, the positions of both modes were comparatively smaller than those generated during vocalization, with maxima in the fitted curves at diameters of 0.36 and 0.82 µm. Both the speaking and singing size distributions showed overall increases in concentration with increasing vocalization loudness. The apparent volume dependence was greater for speaking than for singing; however, this behaviour may be caused by a narrower volume range achieved when singing (see electronic supplementary material, figure S4). Finally, in electronic supplementary material, figure S5, we report mean size distributions according to gender, with no significant differences observed between the size distributions for boys and girls for each activity.

**Figure 6.**
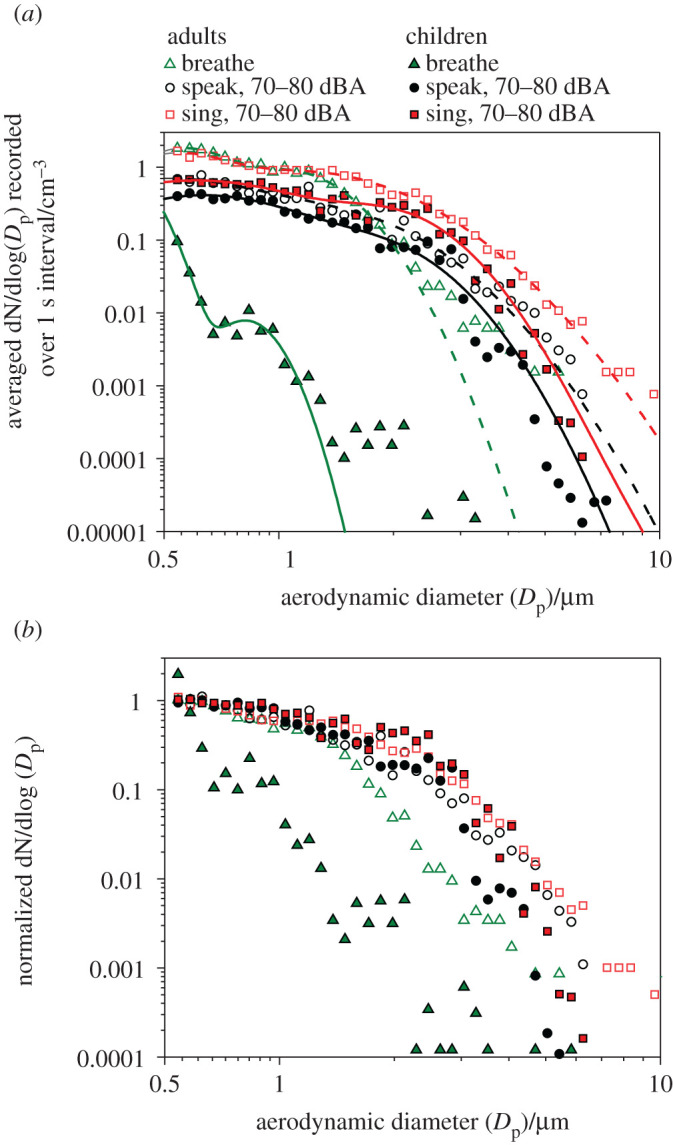
(*a*) Comparison of mean aerosol size distributions generated by children and adult singers when breathing, speaking and singing at 70–80 dBA. Curves of the corresponding colour indicate bimodal lognormal fit of each dataset. (*b*) Mean size distributions for the same cohort and activities, normalized to the mean concentration of the three bins of smallest diameter.

Mean size distributions produced by children breathing, and speaking and singing at a volume of 70–80 dBA are compared in figure 6*a* to those generated from the same activities by a cohort of 25 singers reported by Gregson *et al*. [4]. The overall distribution shapes for each activity are similar for both children and adults. The mean size distribution generated by children while breathing shows a considerably sharper decrease in concentration with increasing diameter than that generated by the adult singers while breathing. By contrast, differences in the generated size distributions between the child and adult cohorts during vocalization are more subtle. During both speaking and singing, children generate fewer particles at smaller diameters than adults, but comparable concentrations at diameters of between 2 and 3 µm. At diameters greater than 3 µm, the concentrations generated by children decrease more sharply than for adults for both speaking and singing. These changes are clearest when distributions are normalized according to the concentrations of the smaller diameter bin and, as shown in figure 6*b*, give rise to substantial differences in the lognormal fitting parameters derived for each subset of participants. The laryngeal mode generated by adults during speaking and singing at 70–80 dBA is best modelled by a broad peak (*σ* of 1.48 ± 0.36 and 1.70 ± 0.09, respectively) at mean *D*_p_ values of 1.34 ± 0.83 and 1.14 ± 0.10 µm, respectively [4]. For children speaking and singing at the same volume, the laryngeal mode is better fitted by a narrower distribution (*σ* of 1.40 ± 0.15 and 1.37 ± 0.10 for speaking and singing, respectively) at larger diameters (1.77 ± 0.47 and 1.93 ± 0.23 µm, respectively).

An important issue not addressed by this study is the relationship between the initial droplet size, relative humidity and the size measured. However, based on our analysis of the sampling of aerosol through the funnel and into the APS [37], we can conclude that the full-size distributions reported here are fully equilibrated in size with sufficient time from exhalation to size measurement. We cannot be confident of the relative humidity at which our size distributions should be reported, and this will be the subject of a future study.

### 3.4. Ventilation measurements and estimates of absolute particle exhalation rates from children and adults while breathing, speaking and singing

In addition to measuring aerosol number concentrations, we have performed measurements of minute ventilation for the entire cohort of children and for a subset of professional adult singers and adults undergoing exercise [34]. Minute ventilation quantifies the mean volume of air expelled from the participant in 1 minute of an activity. Combining the aerosol number or mass concentration with the minute ventilation enables estimation of the absolute exhalation rate of aerosol particle number or mass emitted during a respiratory manoeuvre. The number and mass exhalation rates are expressed in equations (3.1) and (3.2) respectively as

3.1 $$\begin{array}{rl} & \mathrm{number}\;\mathrm{exhalation}\;\mathrm{rate}\;(s^{-1})\\ & \;=\frac{\mathrm{number}\;\mathrm{concentration}\;(l^{-1})\times \mathrm{ventilation}\;(l\;\mathrm{mi}n^{-1})}{60} \end{array}$$

and,

3.2 $$\begin{array}{rl} & \mathrm{mass}\;\mathrm{exhalation}\;\mathrm{rate}\;(\mathrm{ng}\;s^{-1})\\ & \;=\frac{\mathrm{mass}\;\mathrm{concentration}\;(\mathrm{ng}\;l^{-1})\times \mathrm{ventilation}\;(l\;\mathrm{mi}n^{-1})}{60} \end{array}$$

**Figure 7.**
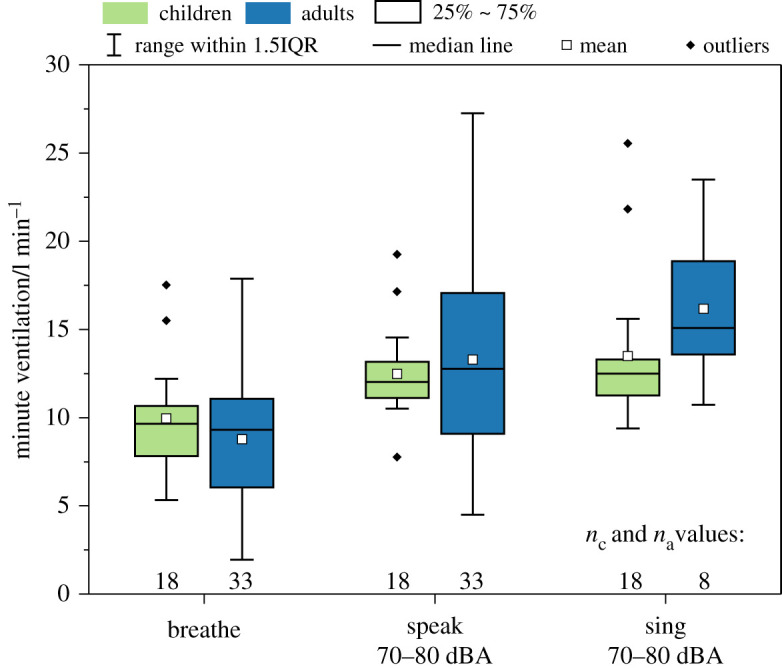
Minute ventilation for the child and adult cohorts while breathing, speaking at 70–80 dBA and singing at 70–80 dBA.

In figure 7, we report minute ventilation for children and adults. Numerical values are provided in table 2. The cohort of children (*n* = 18) had mean minute ventilations of 10.0, 12.5 and 13.5 l min^−1^ during breathing, speaking at 70–80 dBA and singing at 70–80 dBA, respectively. The cohort of adults (*n* = 33 for breathing and speaking, *n* = 8 for singing) had mean minute ventilations of 11.5, 15.9 and 18.6 l min^−1^ during breathing, speaking at 70–80 dBA and singing at 70–80 dBA, respectively. The adults recorded significantly higher minute ventilations than the children for speaking and singing (*p* = 0.004 and *p* = 0.006, respectively), although the absolute differences in minute ventilation are all relatively modest (less than 40%). No significant difference was observed between both cohorts when breathing (*p* = 0.141). Intra-cohort comparisons showed a significantly higher minute ventilation during vocalizing than breathing for both cohorts (children: *p*(breathing different from speaking) = 0.008, children: *p*(breathing different from singing) < 0.001, adults: *p*(breathing different from speaking) < 0.001; adults: *p*(breathing different from singing) = 0.002). No significant difference was observed between speaking and singing for both cohorts (children: *p*(speaking different from singing) = 0.128; adults: *p*(speaking different from singing) = 0.627). In summary, the most notable differences in minute ventilation are observed when comparing breathing with vocalizing for both cohorts.

**Table 2.** Summary of aerosol number and mass exhalation rate obtained from breathing, speaking, and singing at 70–80 dBA for children and adults. Particle exhalation rate (in s^−1^) data correspond to the series of expiratory activities plotted in figure 8*a*, and mass exhalation rate (in ng s^−1^) data correspond to the series of expiratory activities plotted in figure 8*b*. Minute ventilation (in l min^−1^) data correspond to the series of expiratory activities plotted in figure 7. The number of participants for each activity is given for both the children and the wider cohort of adults.

	parameters	activities					
		breathe		speak 70–80 dBA		sing 70–80 dBA	
		children	adults	children	adults	children	adults
particle number exhalation rate (s^−1^)	mean	4.63	8.10	40.2	64.4	86.9	195
	median	4.39	9.80	40.7	60.1	82.9	202
	25%	2.96	3.41	34.9	44.5	63.0	164
	75%	7.36	22.0	54.3	104	174	236
	bottom whisker	1.09	0.477	19.7	11.4	9.95	106
	top whisker	19.5	216	74.5	306	381	325
	*n*	18	33	18	33	18	8
particle mass exhalation rate (ng s^−1^)	mean	0.0040	0.0057	0.097	0.12	0.26	0.31
	median	0.0043	0.0056	0.098	0.11	0.34	0.32
	25%	0.0017	0.0017	0.063	0.071	0.11	0.23
	75%	0.0084	0.017	0.16	0.20	0.82	0.40
	bottom whisker	0.00030	0.00010	0.029	0.030	0.019	0.12
	top whisker	0.034	0.53	0.27	1.3	1.3	0.73
	*n*	18	33	18	33	18	8
minute ventilation (l min^−1^)	mean	9.97	11.5	12.5	15.9	13.5	18.6
	median	9.66	11.8	12.0	15.4	12.5	17.6
	range	(5.33–17.5)	(4.88–20.3)	(7.77 19.3)	(7.35–29.4)	(9.39–25.6)	(13.7–25.7)
	*n*	18	33	18	33	18	8

**Figure 8.**
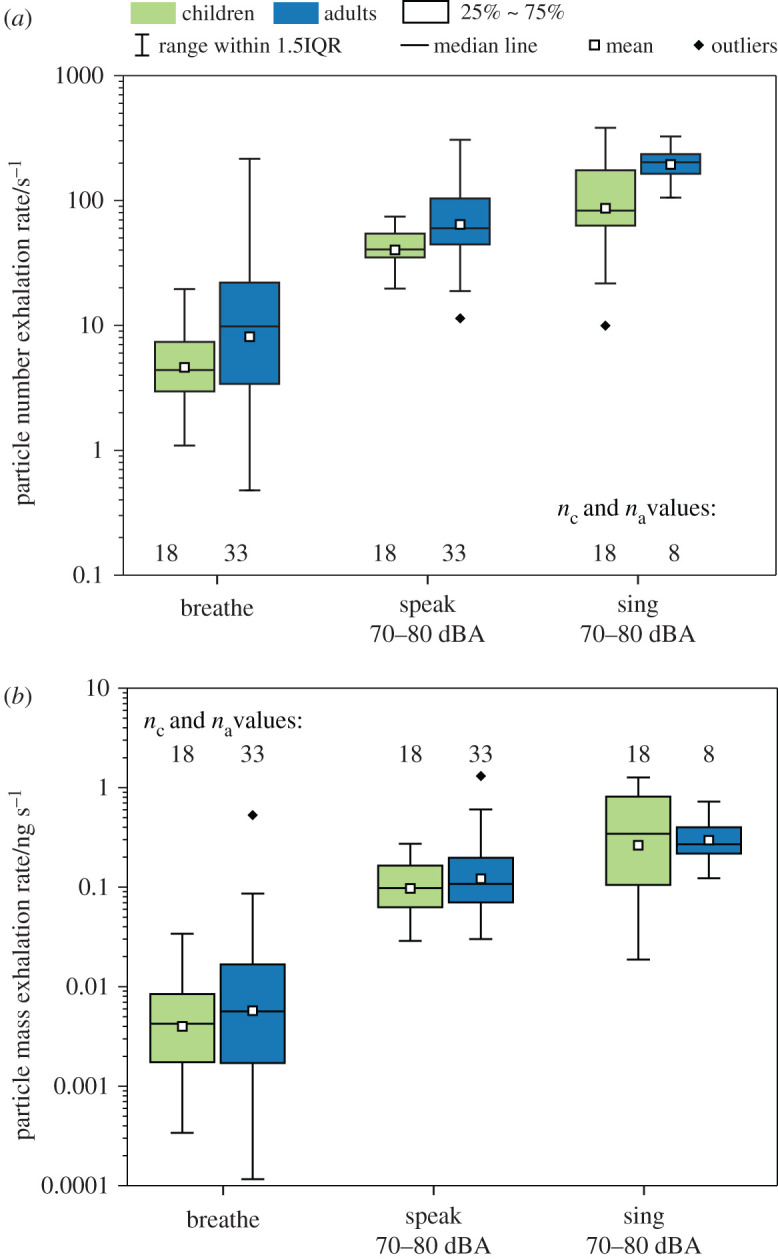
Aerosol (*a*) number and (*b*) mass exhalation rates for breathing, speaking and singing at 70–80 dBA for children and adults.

Figure 8 shows absolute estimates of particle number and particle mass exhalation rates, estimated by combining the measured number or mass concentrations and the minute ventilation data for each participant. Table 2 provides the numerical values. The differences among respiratory activities (breathing versus vocalization) are much larger than the differences between the child and adult cohorts. For both children and adults, speaking generated more aerosol than breathing (6–9 times more by number, *p* < 0.001 for both cohorts; 20–23 times more by mass, *p* < 0.001 for both cohorts), and singing generated modestly more aerosol than speaking at the same loudness level (2–3.4 times more by number, *p* = 0.003 and *p* = 0.013, respectively; 3–3.5 times more by mass, *p* = 0.007 and *p* = 0.006, respectively).

By contrast, children and adults generated similar amounts of aerosol for the same activities. For breathing, the child and adult cohorts emit similarly both in terms of aerosol number (*p* = 0.070) and aerosol mass (*p* = 0.354). For speaking, adults emitted approximately 1.5 times more aerosol than children (*p* = 0.004) by number, but only slightly (around 10%) more by mass (*p* = 0.738). For singing, adults emitted 2.4 times more particles by number than children (*p* = 0.018) but similar amounts in terms of mass (*p* = 0.458). The differences between number and mass arise due to variability in the aerosol size distributions between children and adults.

### 3.5. Effect of ventilation rate and vocalization loudness on aerosol number exhalation rate

**Figure 9.**
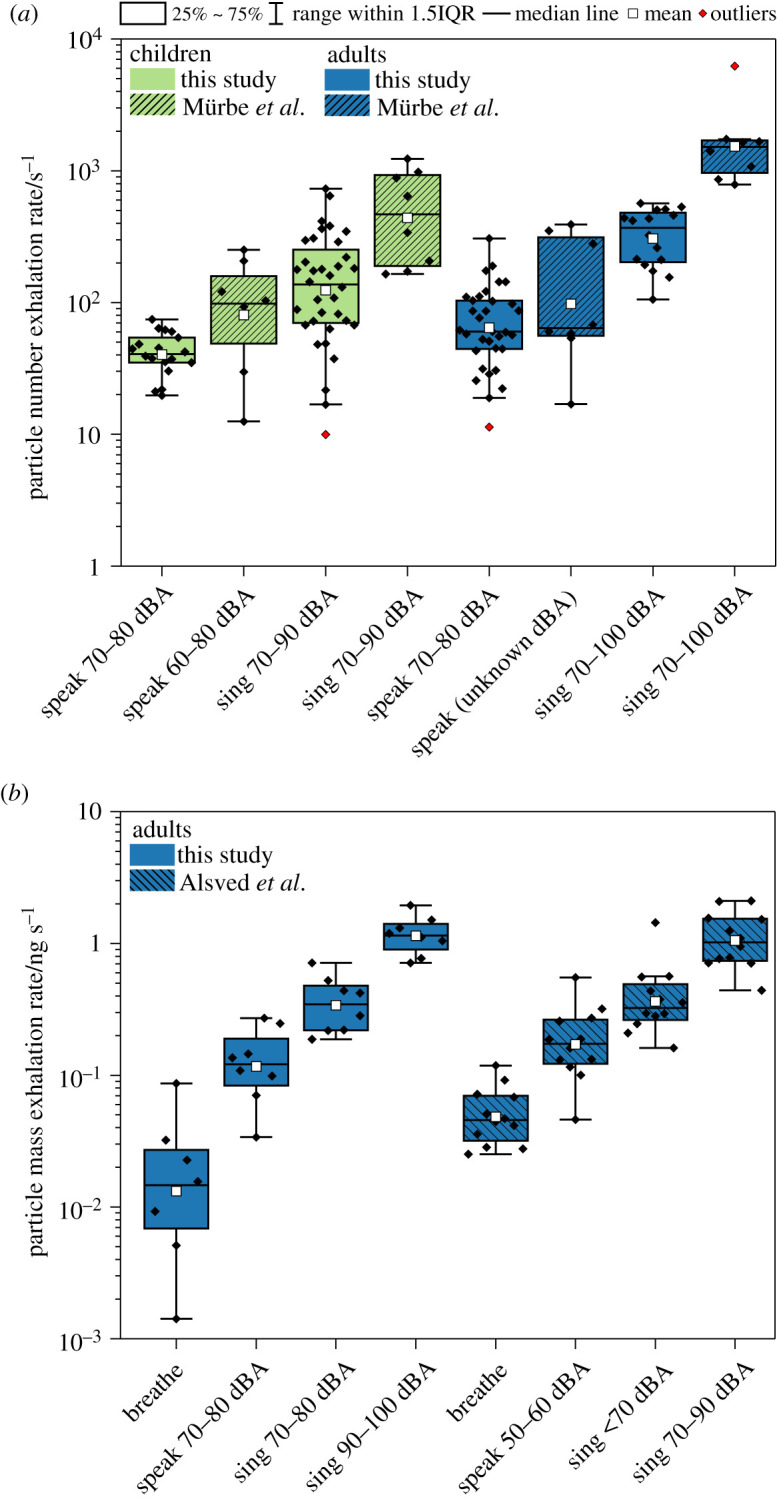
Comparison of absolute exhalations rates from this study with previous estimates. (*a*) Particle number exhalation rates (s**^−^**^1^) from this study and Murbe *et al*. [2,20]. (*b*) Particle mass exhalation rates (ng s**^−^**^1^) from this study and Alsved *et al*. [3]. The diamond dots represent the data points for individual participants.

Our comparisons of absolute particle exhalation rates from speaking and singing for adult and child cohorts are broadly consistent with published studies by Murbe *et al*. [2,20] and Alsved *et al*. [3] as shown in figure 9. The data in the figure are also summarized in electronic supplementary material, table S4. In both, measured particle exhalation rates are scaled to provide absolute estimates of particle exhalation rates. Figure 9*a* compares our values for particle number exhalation rate for both child and adult cohorts while speaking and singing with those of Murbe *et al*. [2,20]. Our values are largely in agreement with theirs, given the differences in approach between the two studies. Our study used a larger cohort, directly measured minute ventilation in each participant, and examined 0.5–20 µm diameter particles. By contrast, Murbe *et al*. used an average ventilation rate of 9.5 l min^−1^ for all participants, a value which is lower than measured rates for all participants in our study, and used an optical particle counter to study a wider aerosol size range (0.3–25 µm in diameter).

Figure 9*b* compares our adult particle mass exhalation rates for breathing, speaking and singing at 70–100 dBA with those reported by Alsved *et al*. [3] demonstrating good agreement in the estimates of the particle mass exhalation rates. The main difference between the two studies is the particle mass exhalation breathing rate for adults. Alsved *et al*. introduced a constant airflow of 15 l min^−1^ into their set-up to ensure fresh air for the participants. This airflow rate was consequently used to calculate an exhalation rate, due to the assumption that air was flowing through the funnel at a rate of 15 l min^−1^. Alsved *et al*. did not consider the minute ventilation of the participants, whereas we directly measured minute ventilation in all participants. Despite not considering the minute ventilation, the use of an airflow rate of 15 l min^−1^ in the calculation of mass exhalation rate serendipitously resulted in rates of the same order of magnitude as those we report. The mean minute ventilations measured in our study were 11.5, 15.9 and 18.6 l min^−1^ for breathing, speaking and singing, respectively (figure 7 and table 2). Thus, the constant airflow of 15 l min^−1^ used by Alsved *et al*. is of similar magnitude to actual minute ventilation for speaking but is larger than that for breathing and smaller than that for singing. Consequently, Alsved *et al*. have likely overestimated their results for breathing and underestimated their results for singing, which may explain higher reported particle mass exhalation rate by Alsved *et al*. for breathing relative to our values.

### 3.6. Effect of ventilation rate and vocalization sound volume on aerosol number exhalation rate

**Figure 10.**
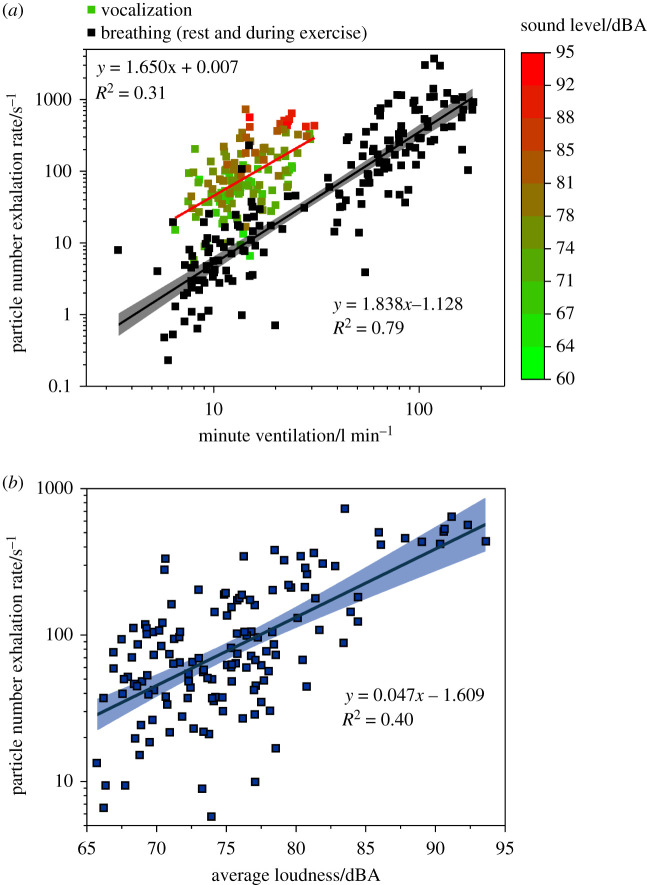
(*a*) Aerosol number exhalation rate determined by multiplying APS measurements (number concentration) and minute ventilation measurements for breathing at rest and during exercise (black squares), and during vocalization (coloured squares) as a function of minute ventilation, either concurrently or in series with aerosol measurements. (*b*) Aerosol number exhalation rates during vocalization activities as a function of concurrently recorded sound level. Linear fits shown by solid lines with 95% confidence interval depicted by shaded region of corresponding colour.

To evaluate the effects of minute ventilation and vocalization loudness on number exhalation rate, we combined aerosol number exhalation rate data from all participants in the PERFORM-2 study for which we also recorded minute ventilation and sound volume. These participants comprised the cohort of 18 children, a representative subset of 8 of the 25 adult singers reported by Gregson *et al*. [4] and a cohort of 25 amateur, intermediate and elite adult athletes [34]. The activities included in this dataset are breathing at rest and during vigorous and very vigorous physical activity, as well as speaking or singing at a range of sound volume levels. The combined data, reported as a function of the minute ventilation measured for the same participant and activity, are shown in figure 10*a*.

Two observations can be drawn from figure 10*a*. First, vocalization generates more particles than breathing for the same minute ventilation rate: number exhalation rates for vocalization are clustered well above those for breathing. This observation is a consequence of vocalization generating an additional mode of aerosol associated with the vocal folds on the larynx relative to breathing. Second, for breathing (no vocalization), number exhalation rates increase with minute ventilation. A linear regression analysis across all breathing-based activities reveals a relatively strong (*R*^2^ = 0.79) correlation between minute ventilation and number exhalation rate. By contrast, a relationship between minute ventilation and number exhalation rate during vocalization is weaker (*R*^2^ = 0.31), although the range of minute ventilation values recorded during vocalization is much smaller than those recorded during exercise of varying intensity.

Several reports have suggested that sound volume is primarily responsible for the number of particles generated during vocalization [1,3,4]. A plot of particle number exhalation rate against the concurrently recorded sound volume levels for a range of vocalization exercises performed by the same cohort of participants is shown in figure 10*b*. Data are fit (*R*^2^ = 0.40) by a linear relationship between sound level and number exhalation rate on a logarithmic scale, in line with our previous study which determined the same relationship for instrument playing [31]. Plots of number concentration against both minute ventilation and sound volume are included in the electronic supplementary material, figure S6. As with number exhalation rate, number concentration also increases with both minute ventilation and sound volume.

## 4. Conclusion

Respiratory viruses and bacteria are exhaled in aerosol particles and droplets when individuals breathe, speak and sing. Increases in particle number and mass concentrations when individuals vocalize at a loud volume compared to when they breathe lead to an increased potential for transmission of respiratory diseases from inhalation of these aerosol particles and droplets [1,4]. In this study, we compare the intensive properties of number and mass concentrations of exhaled aerosols (particles 500 nm–10 µm in diameter) from children (12–14 years of age) and adults (19 to 72 years of age) when breathing, speaking and singing. We also report the extensive properties of exhaled particle number and mass exhalation rates.

Overall, the comparisons of intensive concentrations indicate that children and adults generate similar aerosol concentrations when performing the same activity, but that vocalization generates significantly more aerosol than breathing for both cohorts. Adults and children show similar number concentrations when speaking and singing at 70–80 dBA, and mass concentrations for breathing and singing. A small, but statistically significant, difference in aerosol mass concentration (factor of 2) was observed between the child and adult cohorts during speaking. Within an individual cohort, statistically significant differences between number concentrations when breathing and speaking and between speaking and singing (a factor of 2–3 increase) were observed, similarly mirrored in mass concentration. For both cohorts, the differences between speaking and singing are modest compared to the difference between breathing and speaking. Indeed, a clear dependence on sound volume is observed, with exhaled number and mass concentrations increasing with increasing sound volume for both children and adults when both speaking and singing. For children, statistically significant differences between speaking at 60–70 dBA and 80–90 dBA and between singing at 60–70 dBA and 80–90 dBA are observed. The shapes of the mean size distributions for speaking and singing at a volume of 70–80 dBA are similar for both children and adults. However, the mean size distribution generated by children while breathing shows a considerably sharper decrease in particle number concentration with increasing diameter than generated by adult singers while breathing at particle sizes larger than 1 µm.

To estimate the absolute extensive particle number and mass flux, we compare minute ventilations for adults and children breathing, speaking and singing for the subjects in our study. Consistent with expectations from previous studies, an increase in minute ventilation is observed when vocalizing compared with breathing for both cohorts. Although the adults recorded significantly higher minute ventilations than the children for speaking and singing, the absolute differences are all relatively modest (less than 40%). No significant difference was observed between both cohorts when breathing, nor between speaking and singing.

For absolute particle number exhalation rates, the differences among respiratory activities (breathing versus vocalization) are much larger than the differences between the child and adult cohorts. Indeed, children and adults generated similar amounts of aerosol for the same activities. In broad terms across both cohorts, particle exhalation rates range 3–20 s^−1^ (25–75% range) from breathing, 40–100 s^−1^ from speaking and 70–200 s^−1^ from singing. Mass exhalation rates are 0.002–0.02 ng s^−1^ from breathing, 0.07–0.2 ng s^−1^ from speaking (at 70–80 dBA) and 0.1–0.7 ng s^−1^ from singing (at 70–80 dBA). The scarcity of data on aerosol exhalation rates is hardly surprising when the extremely low values of these number and mass exhalation rates are recognized. For both children and adults, speaking generated more aerosol than breathing (6–9 times more by number, 20–23 times more by mass), and singing generated modestly more aerosol than speaking at the same sound volume level (2–3.4 times more by number, 3–3.5 times more by mass), in line with previous conclusions when reporting the intensive variable of concentration [4]. In addition, as well as clear increases in number and mass exhalation rates with loudness of the vocal activity, the intensive properties of number and mass concentrations increase with exhaled gas flow rate giving strong increases with minute ventilation. Increased exhalation flow velocities at higher minute ventilations lead to increased atomization of respiratory fluids and an increase in the concentrations of particles exhaled (see electronic supplementary material, figure S6). Where comparisons can be made with previously published work, the inter-comparability of data from different studies is remarkable given the challenging nature of such measurements [1–3,20,37].

Although measurements of respirable aerosol number and mass exhalation rates do not provide quantification of viral load or infectious virus, they do provide accurate quantification of the transport medium for viruses. Work by others has reported increased viral load in fine-mode particles smaller than 5 µm in diameter for both SARS-CoV-2 and influenza, the particle size range interrogated by this work [14,15]. As a consequence, the clear dependence of increased aerosol exhalation rate with vocalization compared with breathing and with vocalization at increased sound volume provides insight into the key mitigation measures that can be expected to have the biggest impact on reducing the risk of exposure of individuals to airborne transmission of respiratory viruses, including face coverings and ventilation. Any additional mitigations that reduce the loudness of the vocalization can be also expected to reduce risks. Surprisingly, we demonstrate that both adults and children exhibit similar characteristics in aerosol exhalation rates and, therefore, similar mitigations can be applied to both subject groups.

## Data accessibility

Data underlying the figures and the raw data used in the analysis have been made publicly available in the BioStudies database, https://www.ebi.ac.uk/biostudies/, under accession ID S-BSST754.

The analysed data are provided in electronic supplementary material [38].
